# Changes in Attitudes towards the COVID-19 Vaccine and the Willingness to Get Vaccinated among Adults in Poland: Analysis of Serial, Cross-Sectional, Representative Surveys, January–April 2021

**DOI:** 10.3390/vaccines9080832

**Published:** 2021-07-29

**Authors:** Filip Raciborski, Mateusz Jankowski, Mariusz Gujski, Jarosław Pinkas, Piotr Samel-Kowalik

**Affiliations:** 1 Department of Prevention of Environmental Hazards and Allergology, Medical University of Warsaw, 02-091 Warsaw, Poland; filip.raciborski@wum.edu.pl (F.R.); piotr.samel@wum.edu.pl (P.S.-K.); 2 Centre of Postgraduate Medical Education, School of Public Health, 01-826 Warsaw, Poland; jpinkas@cmkp.edu.pl; 3 Department of Public Health, Medical University of Warsaw, 02-097 Warsaw, Poland

**Keywords:** COVID-19, SARS-CoV-2, vaccinations, vaccine hesitancy, vaccines, Poland

## Abstract

In December 2020, the first coronavirus disease 2019 (COVID-19) vaccine was authorized in the European Union. This study aimed to assess the changes in attitudes towards the COVID-19 vaccine and the willingness to get vaccinated among adults in Poland between January and April 2021. Secondary data analysis was carried out using data obtained from nationally representative cross-sectional surveys (four consecutive waves: January 2021, *n* = 1150; February 2021, *n* = 1179; March 2021, *n* = 1154; April 2021, *n* = 1131) carried out by the Public Opinion Research Center. About 31.3% of individuals declared a lack of willingness to vaccinate against COVID-19 regardless of the study wave. Significant changes (*p* < 0.001) were observed by gender and age. The highest percentage of respondents who declared a lack of willingness to vaccinate against COVID-19 was observed in the youngest age group (18–34 years), 48.5% among males and 45.6% among females. Among individuals over 65 years of age, males significantly more often declared their willingness to be vaccinated than females (*p* < 0.001). The main argument against the COVID-19 vaccine was concern about the potential side effects. Differences in attitudes towards the COVID-19 vaccine in respect of gender and age indicate the need to implement personalized communications to encourage different social groups to vaccinate against COVID-19.

## 1. Introduction

Coronavirus disease 2019 (COVID-19) is a viral infection caused by severe acute respiratory syndrome coronavirus 2 (SARS-CoV-2) [1]. COVID-19 spreads mainly through close contact from person to person via respiratory droplets or aerosols [2,3]. In March 2020, the World Health Organization (WHO) declared the COVID-19 outbreak a global pandemic [4]. As of 21 June 2021, a total of 179,537,236 COVID-19 cases and 3,888,670 COVID-19-related deaths have been reported worldwide [5]. Immunization is the most effective way to protect people against numerous infectious diseases [6]. After the publication of the genetic sequence of the SARS-CoV-2 coronavirus in January 2020, vaccine development was initiated by the pharmaceutical industry [7]. As of March 2020, more than 180 vaccines at various stages of development had been developed by scientists around the world [7]. On 8 December 2020, a 90-year-old British woman became the world’s first person to receive the Pfizer/BioNTech (Pfizer Inc., New York City, U.S./BioNTech SE, Mainz, Germany) COVID-19 vaccine after its approval in the UK [8]. On 11 December 2020, the Food and Drug Administration (FDA) granted an Emergency Use Authorization (EUA) for the Pfizer–BioNTech COVID-19 vaccine in the United States. On 21 December 2020, the European Medicines Agency (EMA) recommended granting conditional marketing authorization for the first COVID-19 vaccine (Pfizer/BioNTech) for authorization in the European Union (EU) [9]. The EMA’s decision was the basis for launching COVID-19 vaccination programs in EU countries.

In December 2020, the EU member states launched the joint purchase of COVID-19 vaccines, with the volume of orders being proportional to the number of inhabitants. Poland is the fifth most populous EU country and ordered 60 million doses of COVID-19 vaccine from six suppliers [10]. On 15 December 2020, the Polish government adopted a resolution on the organization of the National COVID-19 Vaccination Programme [10,11]. The rules of the Programme have changed with the new EMA recommendations (e.g., approval of new COVID-19 vaccines or a change in the age group that may be vaccinated with a given vaccine) and the availability of vaccines from a given manufacturer (Table 1).

Initially, the vaccination schedule specified in the National COVID-19 Vaccination Programme was divided into four stages [11]:

Stage 0—healthcare workers, employees of long-term care facilities and nursing homes, support staff and healthcare administration staff, employees of the State Sanitary and Epidemiological Stations, and parents of premature babies;

Stage I—residents of long-term care facilities and nursing homes, people over 60 years of age in the order of the oldest, teachers, uniformed services;

Stage II—people under the age of 60 with chronic diseases that increase the risk of a severe course of COVID-19 disease; people undergoing diagnostics and treatment, requiring repeated or continuous contact with healthcare facilities; and critical infrastructure workers;

Stage III—entrepreneurs and employees of sectors that have been closed due to the “lockdowns” and mass vaccination of the rest of the adult population.

On 26 December 2020, the first vaccine supplies arrived in Poland, and on 27 December 2020, the first Polish citizen was vaccinated against COVID-19 at the Central Clinical Hospital of the Ministry of Internal Affairs and Administration in Warsaw [12]. The event was broadcast by all mainstream media in Poland and started a nationwide debate on vaccination against COVID-19 [11]. Media communication and statements of medical authorities, politicians, and public opinion leaders are of great importance for building confidence in COVID-19 vaccination and influence attitudes towards the COVID-19 vaccine and willingness to get vaccinated among Poles. Table 1 presents the key facts related to the National COVID-19 Vaccination Programme in Poland.

In Poland, as in the entire EU, vaccinations against COVID-19 are voluntary. The National COVID-19 Vaccination Programme is financed from the state budget, and vaccination is free of charge for all citizens. It is estimated that 70–80% of the country’s population should be vaccinated to achieve herd immunity [13]. High COVID-19 vaccine acceptance is essential for population immunity. Public campaigns, government measures to encourage citizens to vaccinate, and the activity of medical professionals appear to be key activities increasing COVID-19 vaccine acceptance. Wide-spread disinformation and medical fake news on the website have a negative impact on COVID-19 vaccine acceptance [14]. In addition, there is a well-organized anti-vaccine movement in Poland, trying to abolish the mandatory vaccination of people aged 0–19 years, which also undermines confidence in vaccination against COVID-19 [15]. Regular monitoring of attitudes towards the COVID-19 vaccine and willingness to get vaccinated among adults in Poland seems to be a key element of the National COVID-19 Vaccination Programme in Poland. Identification of the groups with the least COVID-19 vaccine acceptance and their motivation against the COVID-19 vaccine allows for the implementation of educational activities to increase COVID-19 vaccine acceptance and counter disinformation.

The aim of this study was to assess the changes in attitudes towards the COVID-19 vaccine and the willingness to get vaccinated among adults in Poland between January and April 2021.

## 2. Materials and Methods

### 2.1. Study Design and Settings

**Figure 1:**
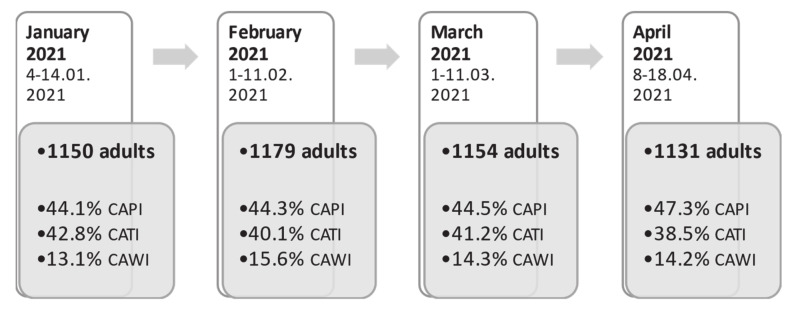
Study flow chart, January–April 2021.

Data were obtained from nationally representative cross-sectional surveys (four consecutive waves: January 2021, *n* = 1150; February 2021, *n* = 1179; March 2021, *n* = 1154; April 2021, *n* = 1131) carried out by the Public Opinion Research Center. The Public Opinion Research Center conducts national cross-sectional surveys on the population’s opinion regarding political and social issues [16]. Data purchased for the purposes of this secondary statistical analysis were driven from cross-sectional questionnaire surveys conducted on representative named-based random samples of Polish residents. Data were collected in parallel using three research techniques: computer-assisted personal interviewing (CAPI), computer-assisted telephone interviewing (CATI) and computer-assisted web interviewing (CAWI) (Figure 1).

### 2.2. Participants

The respondents were adult residents of Poland aged 18–90 years, randomly selected from the PESEL register (population register in Poland). In total, 4611 individuals were included in this study.

### 2.3. Measures

Each of the four survey waves included questions on the COVID-19 pandemic, perception of anti-epidemic measures implemented by public authorities, fear of SARS-CoV-2 coronavirus infection, attitudes towards the COVID-19 vaccine, and the willingness to get vaccinated. Moreover, the respondents were asked about their age, sex, size of the place of residence, and education. For the analysis, only data on the fear of SARS-CoV-2 coronavirus infection as well as attitudes towards the COVID-19 vaccine and the willingness to get vaccinated were used.

Fear of SARS-CoV-2 coronavirus infection was defined using the question: “Are you personally worried about contracting the coronavirus?”. Respondents who indicated “No, I’m not afraid at all” or “No, I’m not afraid” “were classified as those with a lack of fear of SARS-CoV-2 coronavirus infection.

Attitudes towards the COVID-19 vaccine and the willingness to get vaccinated were defined using the question: “Would you like to be vaccinated against COVID-19?”. Respondents who indicated “Definitely not” or “Rather not” “were classified as those who declare negative attitudes towards the COVID-19 vaccine and lack of willingness to vaccinate against COVID-19. Moreover, respondents who declared a lack of willingness to get vaccinated were asked to indicate the reasons for this decision using the question: “Why would you not want to be vaccinated against COVID-19?”.

Moreover, to analyze the potential impact of the dynamics of the COVID-19 pandemic in Poland on the attitudes towards the COVID-19 vaccine, data on the daily number of laboratory-confirmed COVID-19 cases in Poland were collected from epidemiological reports published on the official website of the Polish Ministry of Health [17].

### 2.4. Statistical Analysis

The data were analyzed with SPSS V.27 (Armonk, NY, USA: IBM Corp). Demographic weighting was applied. The distribution of categorical variables was shown by proportions along with 95% confidence intervals. Statistical testing to compare categorical variables was completed using the independent sample chi-squared test. Associations between personal characteristics (age, gender, place of residence, educational level, study wave) and (1) lack of fear of SARS-CoV-2 coronavirus infection and (2) lack of willingness to vaccinate against COVID-19 were analyzed using multivariable logistic regression models. The strength of association was measured by the odds ratio (OR) and 95% confidence intervals (CIs). The level of statistical significance was set at 0.05.

### 2.5. Ethics

This study was carried out following the principles expressed in the Declaration of Helsinki. This study is a secondary statistical analysis and datasets used in this study are anonymous and prevent the identification of any individual study subject by the research team at any stage of the study.

## 3. Results

### 3.1. Characteristics of the Study Population

The demographic characteristics of the study sample are presented in Table 2. The mean age of the respondents ranged between 48.2 and 48.9 years depending on the wave of the study. The percentage of women among the respondents was 52.9%. The share of respondents living in rural areas ranged from 40.2% to 40.6%. In terms of education, the most numerous group comprised people with secondary education, ranging from 31.3% to 31.6% (Table 2).

### 3.2. Dynamics of COVID-19 Pandemic in Poland

**Figure 2:**
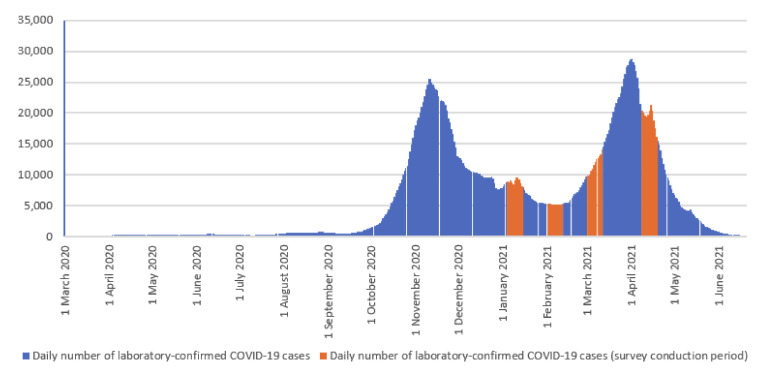
Daily number of laboratory-confirmed COVID-19 cases in Poland, March 2020–June 2021 (rolling 7-day average).

According to the epidemiological reports published on the official website of the Polish Ministry of Health [17], from 4 March 2020 to 20 June 2021, a total of 2.88 million laboratory-confirmed COVID-19 cases and nearly 75,000 COVID-19-related deaths were registered in Poland. It is believed that there were three waves of the COVID-19 pandemic in Poland: March–June 2020, October–December 2020, and March–May 2021. Details are presented in Figure 2.

### 3.3. Fear of SARS-CoV-2 Coronavirus Infection

From January to April 2021, the percentage of respondents who declared lack of fear of SARS-CoV-2 coronavirus infection in Poland fluctuated between 33.1% and 40.3% (*p* < 0.01). The highest percentage of polish inhabitants who declared a lack of fear of SARS-CoV-2 coronavirus infection was observed in February 2021 (Table 2). The percentage of individuals who declared lack of fear of SARS-CoV-2 coronavirus infection significantly differed by gender and age (*p* < 0.001). The highest percentage of respondents (70.2%) who declared lack of fear of SARS-CoV-2 coronavirus infection was observed in February 2021 in the youngest male group (18–34 years) and the lowest (14.2%) was among the female respondents aged 65 years and older. Details are presented in Table 3.

### 3.4. Lack of Willingness to Vaccinate against COVID-19

Between January and April 2021, the percentage of adult inhabitants of Poland who declared lack of willingness to vaccinate against COVID-19 was 31.3%. There were no statistically significant differences (*p* = 0.244) between study waves.

**Figure 3:**
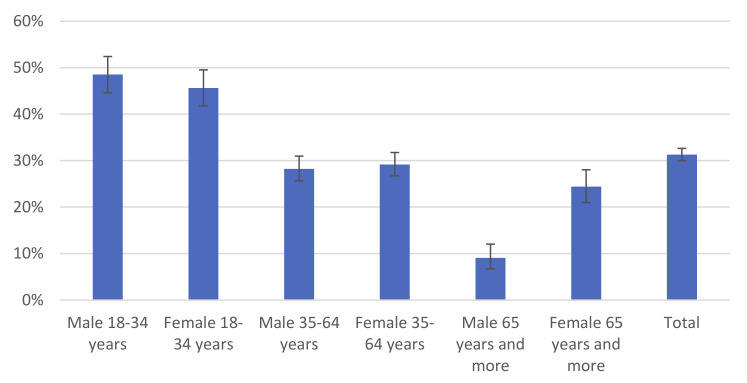
Percentage of respondents who declared lack of willingness to vaccinate against COVID-19 in Poland by gender and age, January–April 2021.

Significant changes (*p* < 0.001) were observed between age groups and by gender. The highest percentage of respondents who declared lack of willingness to vaccinate against COVID-19 (48.5%) was observed in the youngest male group (18–34 years). Similar results (45.6%) were obtained in the group of the youngest females (18–34 years). The lowest (9.1%) percentage of respondents who declared lack of willingness to vaccinate against COVID-19 was among the oldest male group (65 years and more). Details are presented in Figure 3.

### 3.5. Reasons for Negative Attitudes towards the COVID-19 Vaccine and the Lack of Willingness to Get Vaccinated

**Figure 4:**
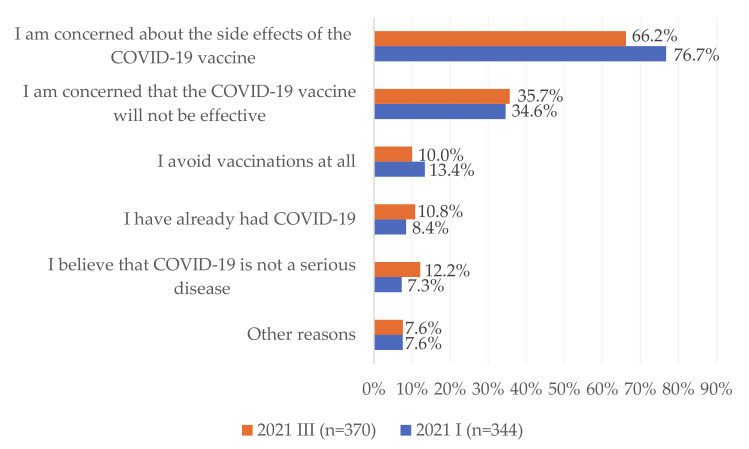
Reasons for negative attitudes towards the COVID-19 vaccine and the lack of willingness to get vaccinated among respondents, who declared that they would not be vaccinated against COVID-19, January–March 2021.

In January and March 2021 reasons for negative attitudes towards the COVID-19 vaccine and the lack of willingness to get vaccinated were examined. The most common causes among respondents were concerns about the potential side effects of the COVID-19 vaccine (71.4%) and concerns about the effectiveness of the COVID-19 vaccine (35.2%) (Figure 4). Between January and March 2021, there was a statistically significant decrease in the percentage of respondents who declared concerns about the potential side effects of the COVID-19 vaccine as the reason for negative attitudes towards the COVID-19 vaccine and the lack of willingness to get vaccinated (from 76.7% to 66.2%; *p* < 0.01). However, the share of people who believed that COVID-19 is not a serious disease increased from 7.3% in January to 12.2% in March 2021 (*p* < 0.05). Details are presented in Figure 4.

### 3.6. Associations between Lack of Fear of SARS-CoV-2 Coronavirus Infection and Negative Attitudes towards the COVID-19 Vaccination

The multivariable logistic regression model for lack of fear of SARS-CoV-2 coronavirus infection showed that respondents aged 18–34 years were 4 times more likely (OR = 4.39; 95%CI: 3.61–5.34) to declare lack of fear of SARS-CoV-2 coronavirus infection compared to those aged 65 years and over. Females compared to males were less likely (OR = 0.63; 95%CI: 0.55–0.71) to declare a lack of fear of SARS-CoV-2 coronavirus infection. Respondents who lived in rural areas (OR = 1.4; 95%CI: 1.1–1.77), those who lived in a city with 20,000 to 49,999 residents (OR = 1.49; 95%CI: 1.13–1.97), as well as respondents who lived in a city with 50,000 to 99,999 residents (OR = 1.49; 95%CI: 1.12–1.99) were more likely to declare lack of fear of SARS-CoV-2 coronavirus infection compared to those who lived in the largest cities (500,000 and more residents). Respondents who were surveyed in February (OR = 1.4; 95%CI: 1.17–1.67) or March (OR = 1.25; 95%CI: 1.04–1.49) were more likely to declare a lack of fear of SARS-CoV-2 coronavirus infection compared to those surveyed in January. Details are presented in Table 4.

According to the multivariable logistic regression model for lack of willingness to vaccinate against COVID-19, age, gender, place of residence, educational level, and study wave were significantly associated with negative attitudes towards the COVID-19 vaccination (Table 4). The highest association was observed in the case of age. Respondents aged 19–34 years were more than 5 times more likely (OR = 5.17; 95%CI: 4.18–6.38) to be unwilling to be vaccinated.

## 4. Discussion

To the best of the authors’ knowledge, this is the most comprehensive study on changes in attitudes towards the COVID-19 vaccine and the willingness to get vaccinated among adults in Poland, carried out during the first months of the National COVID-19 Vaccination Programme. Between January and April 2021, the percentage of adult Poles who declared negative attitudes towards the COVID-19 vaccine and lack of willingness to vaccinate against COVID-19 remained at a stable level (31%), despite the implementation of educational programs, media campaigns, and vaccination promotion by public authorities and medical professionals. The highest percentage of inhabitants of Poland who declared lack of fear of SARS-CoV-2 coronavirus infection was observed in the youngest age group (18–34 years), which led to the fact that in this age group the percentage of individuals who declared lack of willingness to vaccinate against COVID-19 was the highest. Trust in the COVID-19 vaccine and the willingness to get vaccinated increased with age. Among individuals over 65 years of age, males significantly more often declared their willingness to be vaccinated than females. The main argument against the COVID-19 vaccine was concern about the potential side effects.

The EMA’s decision on the conditional marketing authorization for the COVID-19 vaccines in the EU as well as the organization of the National COVID-19 Vaccination Programme meant that every adult Pole had to decide whether they wanted to be vaccinated. In line with the rules of the Programme, the order of vaccination depended on age and occupation. On 14 January 2021, all inhabitants of Poland aged 18–69 could submit a declaration of willingness to get vaccinated (to gauge interest; it was not tantamount to signing up for vaccination, but it allowed a priority vaccination date in the event of vaccination for a given Stage of the Programme). As of 10 May 2021, all persons over the age of 18 could schedule an appointment for the COVID-19 vaccine. As of 21 June 2021, approximately 52% of adult Poles had taken at least one dose of the COVID-19 vaccine [18]. Data from the National COVID-19 Vaccination Programme show that the interest in COVID-19 vaccinations decreased over the following weeks, and the commencement of the vacation period (July–August) may significantly affect the dynamics of vaccination. For this reason, it is important to identify groups who declared negative attitudes towards the COVID-19 vaccine and lack of willingness to vaccinate against COVID-19, to apply personalized communication tailored to the individual needs of people declaring negative attitudes towards COVID-19 vaccination [19,20].

Our study shows that the percentage of respondents who declared a lack of fear of SARS-CoV-2 coronavirus was related to the daily number of laboratory-confirmed COVID-19 cases. The highest percentage of respondents who declared lack of fear of SARS-CoV-2 coronavirus infection was observed in February, i.e., between the second and third waves of the COVID-19 pandemic in Poland. The highest percentage of inhabitants of Poland who declared lack of fear of SARS-CoV-2 coronavirus infection was observed among those aged 18–34 years, which results from the fact that the risk of a severe course of COVID-19 and death is the lowest among young adults, and significantly increases after the age of 60 [1,2]. It can be assumed that this was the most important factor contributing to the fact that the greatest willingness to vaccinate against COVID-19 was observed among respondents over 65 years of age. Interestingly, among people over 65 years the percentage of Poles who declared lack of willingness to vaccinate against COVID-19 was higher among females than among males, which suggests that despite fear of infection, older women do not want to be vaccinated against COVID-19. Further research is needed to explain this phenomenon.

In this study, the highest percentage of inhabitants of Poland who declared a lack of willingness to vaccinate against COVID-19 was observed among those aged 18–34 years. Importantly, with the increasing daily number of new COVID-19 cases, the percentage of people who declared a lack of willingness to vaccinate against COVID-19 has decreased. It can be assumed that the communication strategy on vaccination against COVID-19 so far has been the most effective in this age group.

We observed that the most common reasons for negative attitudes towards the COVID-19 vaccine and the lack of willingness to get vaccinated among respondents were concerns about the potential side effects of the COVID-19 vaccine and concerns about the effectiveness of the COVID-19 vaccine. Suspension of the use of the AstraZeneca COVID-19 vaccine in selected EU countries has led to increased concerns about the potential side effects of COVID-19 vaccines [21]. This hypothesis is in line with the study by Rzymski et al., who observed that among adult Poles the highest level of trust was observed for the mRNA COVID-19 vaccines [22]. Despite the official position of the European Medicines Agency, which defined blood clotting as a “very rare” side effect of the AstraZeneca COVID-19 vaccine, misinformation about the COVID-19 vaccine has been disseminated on social media [23]. Moreover, the real-world evidence from countries where mass vaccination resulted in a significant decrease in the number of new COVID-19 cases did not constitute a sufficient argument for people who declared negative attitudes towards the COVID-19 vaccine. In addition, medical fake news about COVID-19 vaccines has been disseminated online by the anti-vaccine movement with well-organized social media channels [15,23]. During the implementation of the National COVID-19 Vaccination Programme, the anti-vaccination movement in Poland regularly organized public gatherings and protests against COVID-19 vaccines. We can hypothesize that the activity of the anti-vaccine movement and its supporters on the Internet was the main reason why, despite widespread and free access to the COVID-19 vaccine, still almost one-third of adult Poles declared a lack of willingness to vaccinate against COVID-19. We can hypothesize that spreading medical fake news on the Internet may shape negative attitudes towards the COVID-19 vaccine among the youngest age groups. Moreover, lack of fear of SARS-CoV-2 infection among the youngest groups (mostly due to the mild/moderate COVID-19 course in this age group) may discourage young people from vaccinating against COVID-19.

Due to the fact that previous studies on the attitudes of Poles towards vaccination against COVID-19 have been limited to selected social groups, including cancer patients, ophthalmology residents, and healthcare workers, or based on a web-based questionnaire, the results of our study are difficult to compare due to different populations and study methodologies [24,25].

Our study provides information on target groups that should now be a priority for the National COVID-19 Vaccination Programme. Educational and communication activities should be targeted at people aged 18–34, where the percentage of Poles who declared lack of willingness to vaccinate against COVID-19 was the highest. It seems that social media should be selected as the main communication channel targeting this age group [19,20]. Moreover, females aged over 65 years should be encouraged to vaccinate against COVID-19, especially by family doctors, due to the increase in the percentage of people who declared negative attitudes towards the COVID-19 vaccine between March and April. Moreover, reasons for negative attitudes towards the COVID-19 vaccine and the lack of willingness to get vaccinated among respondents declared by the respondents point to the need to constantly present scientific data on the safety and effectiveness of COVID-19 vaccines and to proactively counteract fake news on COVID-19 vaccines. Physicians and policymakers should adapt the message to different age groups. In addition, physicians should regularly communicate new scientific findings on vaccine safety and effectiveness, especially in the case of protecting against new variants of the coronavirus. Moreover, the content of the message should be formulated in a simple and understandable way to reduce the barriers to understanding the information depending on the individual level of health literacy.

This study has several limitations. First, we did not assess attitudes towards particular types of vaccines. The percentage of people who declare willingness to vaccinate against COVID-19 may differ depending on the type of vaccine, which should be verified in future studies. Secondly, the research methodology used in this study (secondary analysis), did not allow for an assessment of the effectiveness of individual methods of communication about COVID-19 vaccines and their impact on attitudes towards the COVID-19 vaccine. Thirdly, attitudes towards the COVID-19 vaccine were defined based on self-reported data on willingness to vaccinate against COVID-19, so we cannot exclude the possibility of recall bias. Fourth, the potential selection bias during fieldwork is also a limitation of our study. In Poland, a decline in the crude response rate has been observed for many years [26]. This may affect the presented estimations.

## 5. Conclusions

Between January and April 2021, despite the implementation of educational programs, media campaigns and vaccination promotion by public authorities and medical professionals, almost one-third of adult inhabitants of Poland declared a lack of willingness to vaccinate against COVID-19. The willingness to get vaccinated increased with the age. Among individuals over 65 years of age, males significantly more often declared their willingness to be vaccinated than females. The main argument against the COVID-19 vaccine was concern about the potential side effects. Differences in the attitudes towards the COVID-19 vaccine in respect of gender and age indicate the need to implement personalized communication and message segmentation to encourage different social groups to vaccinate against COVID-19.

## Figures and Tables

**Table 1:** National COVID-19 Vaccination Programme in Poland—key facts.

Date	Event/Decision Regarding Vaccination against COVID-19
8 December 2020	The world’s first person received the Pfizer/BioNTech COVID-19 vaccine after its approval in the UK
15 December 2020	The Polish government adopted a resolution on the organization of the National COVID-19 Vaccination Programme
21 December 2020	EMA’s decision on the conditional marketing authorization for the first COVID-19 vaccine (Pfizer/BioNTech) in the EU
27 December 2020	The first Polish citizen was vaccinated against COVID-19 as part of the National COVID-19 Vaccination Programme; mass vaccination of healthcare workers (Stage 0)
29 December 2020	Public vaccination of physicians and politicians, including representatives of the Polish parliament and the Ministry of Health
6 January 2021	EMA’s decision on the conditional marketing authorization for the second COVID-19 vaccine (Moderna) in the EU
14 January 2021	All citizens aged 18–69 could submit a declaration of willingness to get vaccinated (to gauge interest; it was not tantamount to signing up for vaccination, but it allowed a priority vaccination date in the event of vaccination for a given Stage of the Programme)
15 January 2021	Allowing people over 80 years of age to register for vaccination
18 January 2021	Vaccination of residents of long-term care facilities and nursing homes (approximately 70,000 people)
22 January 2021	Allowing people over 70 years of age to register for vaccination
25 January 2021	Vaccination of people over 60 years of age (Stage I)
29 January 2021	EMA’s decision on the conditional marketing authorization for the third COVID-19 vaccine (AstraZeneca (AstraZeneca plc, Cambridge, UK)) in the EU
12 February 2021	Vaccination of teachers (approximately 270,000 teachers out of 440,000 eligible)
3 March 2021	Austria suspended the use of one batch of AstraZeneca COVID-19 vaccine after two persons suffered blood clots after vaccination
6 March 2021	Time interval for second dose of COVID-19 vaccine extended (AstraZeneca up to 12 weeks; Pfizer/BioNTech and Moderna up to 42 days)
10 March 2021	Amendment of the ordinance—COVID-19 survivors can be vaccinated not earlier than three months from the date of obtaining a positive result of the RT-PCR test
11 March 2021	EMA’s decision on the conditional marketing authorization for the single-dose COVID-19 vaccine (Johnson & Johnson) in the EU; Denmark and Norway suspend AstraZeneca COVID-19 vaccine over blood clot concerns
23 March 2021	Vaccination of people aged 60–64 years
24 March 2021	Vaccination of uniformed services
9 April 2021	Dentists, nurses, midwives, paramedics, physiotherapists, and pharmacists authorized to qualify patients for vaccination against COVID-19
19 April 2021	Starting vaccinations at the Common Vaccination Centers (large points organized in central places in towns and villages or at sports stadiums)
20 April 2021	The possibility of vaccinating any person over 18 years of age if there is a risk that the vaccine will be wasted
23 April 2021	Public vaccination (AstraZeneca) of the Polish Prime Minister
26 April 2021	Public vaccination of the Polish President
1–3 May 2021	Mobile vaccination points (single-dose Johnson & Johnson COVID-19 vaccine) in major Polish cities during the national holiday
10 May 2021	All persons over the age of 18 can schedule an appointment for the COVID-19 vaccine
17 May 2021	Time interval for second dose of COVID-19 vaccine shortened (AstraZeneca from 84 days to 35 days; Pfizer/BioNTech and Moderna from 42 to 35 days); COVID-19 survivors can be vaccinated not earlier than 30 days from the date of obtaining a positive result of the reverse transcription polymerase chain reaction (RT-PCR) test; allowing people aged 16–18 years to register for vaccination
31 May 2021	A total of 20 million doses of the COVID-19 vaccine had been administered in Poland
1 June 2021	Fully vaccinated people have obtained the EU Certificate of Vaccination against COVID-19
7 June 2021	Allowing people aged 12–15 years to register for vaccination

**Table 2:** Characteristics of the study population.

Study Date (Wave)	January 2021	February 2021	March 2021	April 2021
*n*	1150	1179	1154	1131
Age (years)				
mean ± SD	48.7 (17.8)	48.8 (17.7)	48.2 (18.2)	48.2 (17.8)
≤34	27.6%	27.6%	27.0%	26.9%
35–64	50.4%	50.5%	51.0%	51.0%
65+	22.0%	22.0%	22.0%	22.1%
Gender				
Male	47.1%	47.2%	47.1%	47.1%
Female	52.9%	52.8%	52.9%	52.9%
Place of residence				
rural	40.5%	40.2%	40.6%	40.6%
city below 20,000 residents	12.7%	15.9%	14.0%	13.1%
city from 20,000 to 49,999 residents	12.2%	11.6%	11.7%	11.1%
city from 50,000 to 99,999 residents	9.2%	8.5%	9.8%	10.9%
city from 100,000 to 499,999 residents	14.6%	14.5%	13.9%	14.5%
city 500,000 and more residents	10.8%	9.2%	10.0%	9.9%
Educational level				
primary education	16.8%	16.3%	16.4%	16.4%
vocational education	23.7%	23.4%	23.4%	23.4%
secondary education	31.3%	31.6%	31.6%	31.5%
higher education	28.2%	28.7%	28.5%	28.6%

**Table 3:** Percentage of respondents who declared lack of fear of SARS-CoV-2 coronavirus infection in Poland by gender and age, January–April 2021.

Study Date (Wave)	January 2021		February 2021		March 2021		April 2021		*p*-Value
	%	95%CI	%	95%CI	%	95%CI	%	95%CI	
Male 18–34 years	57.8	(50.2–65)	70.2	(62.3–77)	59.7	(52.2–67.5)	55.1	(47.3–62.6)	<0.05
Female 18–34 years	49.0	(41.3–57.3)	55.6	(48.3–62.8)	53.7	(46–61.5)	42.0	(34.5–50.5)	0.078
Male 35–64 years	35.5	(29.7–41.5)	45.3	(39.6–51.1)	37.5	(31.8–43.1)	36.2	(30.6–41.9)	0.061
Female 35–64 years	21.6	(17.3–26.2)	27.2	(22.5–32.4)	30.6	(25.7–35.9)	29.4	(24.5–34.7)	<0.05
Male 65 years and more	25.0	(18.1–33.5)	23.3	(16.2–31)	28.3	(20.4–37.1)	26.5	(18.4–35.5)	0.846
Female 65 years and more	15.2	(9.7–21.7)	22.4	(16.2–30.2)	18.8	(13–25.7)	21.8	(15.8–28.9)	0.361
Total	33.1	(30.5–35.9)	40.3	(37.5–43.1)	37.6	(34.8–40.4)	35.0	(32.3–37.8)	<0.01

**Table 4:** Associations between lack of fear of SARS-CoV-2 coronavirus infection and negative attitudes towards the COVID-19 vaccination—multivariable logistic regression model.

Variable	Model for Lack of Fear of SARS-CoV-2 Coronavirus Infection			Model for Lack of Willingness to Vaccinate against COVID-19		
	*p*-Value	OR	95%CI OR	*p*-Value	OR	95%CI OR
Age						
≤34	<0.001	4.39	(3.61–5.34)	<0.001	5.17	(4.18–6.38)
35–64	<0.001	1.69	(1.41–2.02)	<0.001	2.08	(1.71–2.53)
65+	Ref.	Ref.	Ref.	Ref.	Ref.	Ref.
Gender						
Male	Ref.	Ref.	Ref.	Ref.	Ref.	Ref.
Female	<0.001	0.63	(0.55–0.71)	*p* < 0.01	1.21	(1.06–1.38)
Place of residence						
rural	<0.01	1.4	(1.1–1.77)	<0.001	2.42	(1.84–3.18)
city below 20,000 residents	0.157	1.22	(0.93–1.59)	<0.001	1.94	(1.43–2.65)
city from 20,000 to 49,999 residents	<0.01	1.49	(1.13–1.97)	<0.001	2.33	(1.7–3.19)
city from 50,000 to 99,999 residents	<0.01	1.49	(1.12–1.99)	<0.001	2.26	(1.63–3.13)
city from 100,000 to 499,999 residents	0.053	1.3	(1–1.7)	<0.01	1.61	(1.18–2.19)
city 500,000 and more residents	Ref.	Ref.	Ref.	Ref.	Ref.	Ref.
Educational level						
primary education	0.51	1.07	(0.87–1.33)	<0.001	1.76	(1.41–2.19)
vocational education	0.796	1.03	(0.85–1.24)	<0.001	1.77	(1.46–2.16)
secondary education	0.289	1.09	(0.93–1.28)	<0.001	1.51	(1.27–1.79)
higher education	Ref.	Ref.	Ref.	Ref.	Ref.	Ref.
Study wave						
January 2021	Ref.	Ref.	Ref.	Ref.	Ref.	Ref.
February 2021	<0.001	1.4	(1.17–1.67)	0.08	1.18	(0.98–1.41)
March 2021	<0.05	1.25	(1.04–1.49)	0.239	1.12	(0.93–1.34)
April 2021	0.303	1.1	(0.92–1.32)	0.937	1.01	(0.84–1.21)

## Data Availability

The datasets generated during and/or analyzed during the current study are available from the corresponding author on reasonable request.
